# Digit ratio (2D:4D) in women and men with lung cancer

**DOI:** 10.1038/s41598-020-68239-0

**Published:** 2020-07-09

**Authors:** Anna Kasielska-Trojan, J. T. Manning, A. Antczak, A. Dutkowska, W. Kuczyński, A. Sitek, B. Antoszewski

**Affiliations:** 1 Plastic, Reconstructive and Aesthetic Surgery Clinic, Institute of Surgery, Medical University of Lodz, Kopcinskiego 22, 90-153 Lodz, Poland; 2 Applied Sports, Technology, Exercise, and Medicine (A-STEM), Swansea University, Swansea, UK; 3 Department of General and Oncological Pulmonology, Medical University of Lodz, Lodz, Poland; 4 Department of Sleep Medicine and Metabolic Disorders, Medical University of Lodz, Lodz, Poland; 5 Department of Anthropology, Faculty of Biology and Environmental Protection, University of Lodz, Lodz, Poland

**Keywords:** Diseases, Risk factors

## Abstract

A prenatal sex steroid environment of high prenatal testosterone and low prenatal oestrogen inhibits lung development and may predispose individuals to be vulnerable to lung disease in later life. Therefore, the aim of this report was to investigate whether there is an association between right and left 2D:4D (biomarker of prenatal sex steroids exposure) and primary lung cancer in women and men. Also, we considered the relationship between right–left 2D:4D (Δ2D:4D, a negative correlate of high prenatal testosterone and low prenatal oestrogen) and the age of lung cancer diagnosis. The study included 109 patients (61 men) with lung cancer and 197 controls (78 men). In the study we found that: (i) women with lung cancer have lower 2D:4D compared to controls (the effect was independent of smoking), (ii) among women with cancer, age at diagnosis was positively related to 2D:4D, i.e. women with masculinized 2D:4D present earlier with the cancer than women with feminized 2D:4D, (iii) among men with lung cancer, those with the most aggressive form (small-cell lung cancer) had masculinized (low) Δ2D:4D compared to those with the less aggressive form (non-small cell lung cancer). The data suggests that masculinized right 2D:4D and Δ2D:4D are associated with a predisposition to lung cancer and/or the more aggressive forms of lung cancer.

## Introduction

Among both women and men, lung cancer is the leading cause of cancer death and in most nations, it is the most frequent neoplasm among men^1,2^. The incidence of lung cancer is both age- and sex-dependent. With regard to age, rates are low in people younger than 40 years but with increases up to the age of 75–80 years. However, with regard to sex, lung cancer rates have tended to show a decline in men but an increase among women. An important causal factor in these patterns is smoking practices^3–5^. The comparisons between those that smoke and never-smokers regularly show 20 to 50-fold increases in risk for the former. Moreover, duration of smoking is a strong correlate of lung cancer risk^6^ and sex dependent changes in mortality reflects changes in rates of smoking among age-cohorts of women and men^7,8^. However, there are risk factors other than smoking and these include various single nucleotide polymorphisms, family history, diet, alcohol, chronic lung diseases, occupational factors, air pollution and hormonal factors^9–13^.

With regard to hormonal factors, postnatal effects of endogenous sex steroids may have an influence on lung cancer risk. Receptors for oestrogen and progesterone are expressed in lung cells, of both normal and lung cancer lines. Oestradiol causes proliferation of lung cancer cells as do combinations of oestrogen and progesterone. The latter combination facilitates secretion of vascular endothelial growth factors and increases numbers of progenitor tumour cells^14,15^. Local production of oestrogens in the lung, either by lung cells or by infiltrating macrophages and other inflammatory cells, may be a significant source of oestrogen that could drive the tumour process, independent of reproductive tissues. Oestrogen produced as a result of pulmonary inflammation may be an important driver of the pro-tumour consequences of chronic inflammation in the lung^16^. However, in contrast to the effect of endogenous sex steroids, exogenous oestrogen hormone replacement therapy may have no effect or a protective effect on lung cancer risk in women^17,21^. Nevertheless, sex hormones play a key role in the new therapeutic strategies in lung cancer^22,23^. Concerning prenatal sex steroids influence on lung development, different expression of oestrogen receptors β (ERβ) mRNA compared to oestrogen receptors α (ERα) mRNA was found in human lung tissue during foetal development^24^. In experimental studies on mice with ERβ knockout there were lung abnormalities in the number of alveoli and reduction in expression of key regulators of surfactant homeostasis^25^. However, most links between sex steroids and lung cancer relate to postnatal effects, there are no clinical studies on associations between lung cancer and biomarkers of prenatal sex steroids.

The relative lengths of the second digit and fourth digit (digit ratio or 2D:4D) is sexually dimorphic (2D:4D males < 2D:4D females). Manning et al. have suggested that 2D:4D is a biomarker of prenatal sex steroids exposure—low 2D:4D correlates with high prenatal testosterone and low oestrogens, while high 2D:4D results from low foetal testosterone and high oestrogens^26–28^. The associations between 2D:4D and prenatal sex steroids are greater for the right hand and right–left 2D:4D (Δ r–l 2D:4D) is also a negative correlate of high prenatal testosterone and low prenatal oestrogen^28–30^. Experimental evidence for the suggestions of Manning et al. have been reported in mouse models by Zheng and Cohn and in rats by Auger et al. They found that sex differences in 2D:4D (males < females) are determined by a balance of prenatal testosterone to oestrogen in a narrow time window of foetal digit development^31,32^.

Some researchers suggest that 2D:4D ratio can serve as a biomarker for cancers that show a sex dependent pattern on disease occurrence, progression or prognosis. A recent review and meta-analysis concerning 2D:4D associations with cancer revealed that the sex hormone environment during early development may be associated with cancer risk later in life. Low 2D:4D was associated with prostate cancer, gastric cancer, and brain tumours risk, while high 2D:4D, with breast cancer and cervical dysplasia. Additionally, higher 2D:4D is associated with breast cancer risk and with younger age of breast cancer and brain tumour diagnosis^33,34^.

Here we ask whether 2D:4D is related to lung cancer. The question of whether 2D:4D is associated with lung cancer is essentially one which relates to the influence of the prenatal balance of testosterone and oestrogen on lung development. It does not relate to the effect of post-natal sex steroids on the aetiology of lung cancer because 2D:4D is not closely correlated with post-natal concentrations of testosterone and oestrogen^35^. There are a number of parameters in lung development that are sexually dimorphic suggesting a role for prenatal testosterone and oestrogen. With regard to the latter, Mendelson et al. (1980) reported an oestrogen-binding component in human foetal lung tissue. The maturation of lungs during foetal development is more rapid in female foetuses than male foetuses. Moreover, the onset of surfactant synthesis occurs later in the male foetus. These differences are mediated by a balance of testosterone and oestrogen in the foetus, with the former having an inhibitory effect and the latter a stimulatory effect on lung development^36,37^.

A prenatal sex steroid environment of high testosterone and low oestrogen inhibits lung development and may predispose individuals to be vulnerable to lung disease in later life. Therefore, we hypothesise that low 2D:4D, particularly of the right hand, may be associated with lung cancer in both sexes and that low Δ2D:4D may be associated with a greater propensity to develop lung cancer. The aim of this report was to investigate an association between right and left 2D:4D and primary lung cancer in women and men and the relationship between Δ2D:4D and the age of lung cancer diagnosis.

## Methods

Participants were recruited from a General and Oncologic Pulmonology Clinic in a University Hospital. All consecutive patients with diagnosed lung cancer who were qualified for chemotherapy due to the advanced stage of the disease and/or other clinical indications were included, regardless of histological type of the cancer. During a 1-year period 109 (61 men and 48 women) patients met the study criteria and gave written informed consent for participation in this study. The protocol of the study included a clinical questionnaire (age, age of diagnosis, histopathological type of the cancer, stage of the disease, concomitant diseases, history of smoking and occupational exposure, family history of cancers) and anthropometric measurements. All measurements were done before chemotherapy implementation.

Controls (119 women (the mean age 52.57 ± 15.02) and 78 men (the mean age 51.13 ± 15.85) were recruited from Plastic Surgery Clinic patients with no cancer in anamnesis, without congenital abnormalities and hormonal disturbances. Those who reported hand injuries were excluded from the study (2 male patients).

The participants completed a clinical questionnaire and anthropometric measurements were performed. All the participants were White (based on the data reported in the questionnaire). The protocol was agreed by the Bioethical Committee of the Medical University of Lodz (RNN/368/18/KE). All methods were performed in accordance with the relevant guidelines and regulations.

### Lung cancer patients’ characteristics

The studied group included 61 men (the mean age 64.44 ± 7.75 years) and 48 women (67.23 ± 9.61 years). In men the mean age of lung cancer diagnosis was 63.9 ± 7.75 years and in women—66.79 ± 10 years. Among men the following histopathological types of lung cancer (lc) were found: 14 (22.9%) small cell lc, 23 (37.7%)—squamous cell lc, 19 (31.1%)—adenocarcinoma, 5 (8.2%)—large cell lc and other types, while in women: 11 (22.9%) small cell lc, 14 (29.2%)—squamous cell lc, 18 (37.5%)—adenocarcinoma, 5 (10.4%)—large cell lc and other types. The distribution was similar in women and men (Chi^2^ = 1.04; df = 3; p = 0.79). Staging of lung cancer according to the American Joint Committee on Cancer (AJCC) in men group was: I-1, II-3, III-19 and IV-38 and in female: I-1, II-2, III-22, IV-23. Three men suffered from additional primary cancer: 2- prostate and 1- liver and 7 had a family history of lung cancer (parent, grandparent, brother/sister, child). Four women had additional primary cancer—breast cancer and 6 had a family history of lung cancer. In total, 56 (91.8%) men and 44 (91.7%) women were smokers.

### Measurements

Eight measurements were taken from patients and controls: body height, waist and hip circumferences, second and fourth digits’ lengths (2D and 4D) (right (R) and left hand (L)) and body weight. On the basis of the these parameters the following variables were calculated: BMI (body weight [kg]/(height)^2^ [m^2^]), WHR (waist circumference [cm]/hip circumference[cm]), 2D:4D for the right (R) and left (L) hand (2D length [mm]/4D length [mm]) (2D:4D R, 2D:4D L) and right minus left 2D:4D (Δ2D:4D). All measurements were made directly with GPM anthropological instruments (sliding calliper, anthropometer, measuring tape). Measurements were performed on the palmar side of the hand using anthropometric points lying on the digit axis: pseudophalangion—a point in the finger metacarpophalangeal crease, dactylion—the most distal point on the fingertip^38^.

Body size ratios (BMI and WHR) were excluded from the study protocol as patients with advanced lung cancer might have current weight loss/cachexia related to the disease.

### Statistical analysis

Logistic regression was used to evaluate the relationship between the 2D:4D and the risk of lung cancer. 2D:4D of each hand was evaluated in separate models, each time adjusted for smoking. Wald test was used for checking regression parameters’ relevance. We also considered whether 2D:4D correlates with the age of lung cancer onset and if there was a correlation between 2D:4D and smoking in female and male groups. *p* < 0.05 was accepted as a level of significance. The correlation between histological type of lung cancer and 2D:4D in women and men was tested with t test. The population effect size for intra-group differences was evaluated with Hedges' g. Cohen (1992) suggested the following interpretation of Hedges' g effect size: > 0.2—weak, > 0.5—medium, > 0.8—strong effect^39^.

## Results

### 2D:4D and the risk of lung cancer

**Figure 1:**
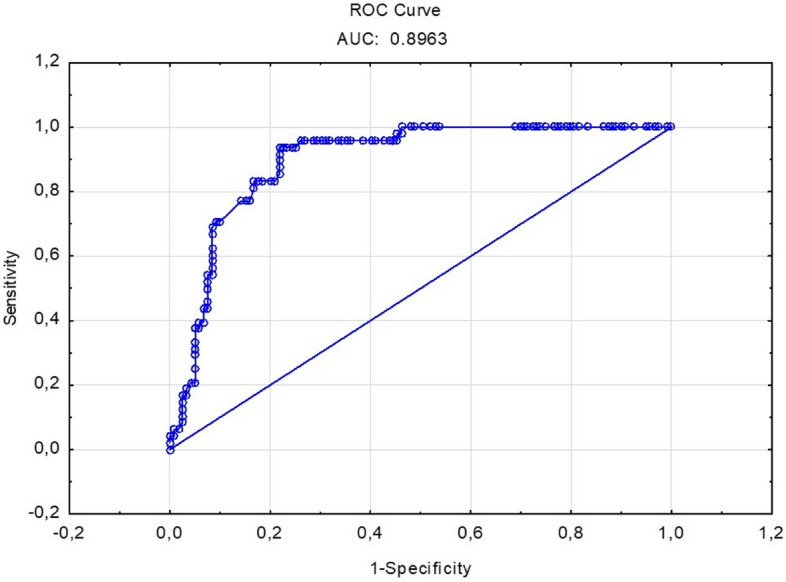
ROC curve for the model predicting probability of lung cancer in women on the basis of smoking and right 2D:4D.

**Figure 2:**
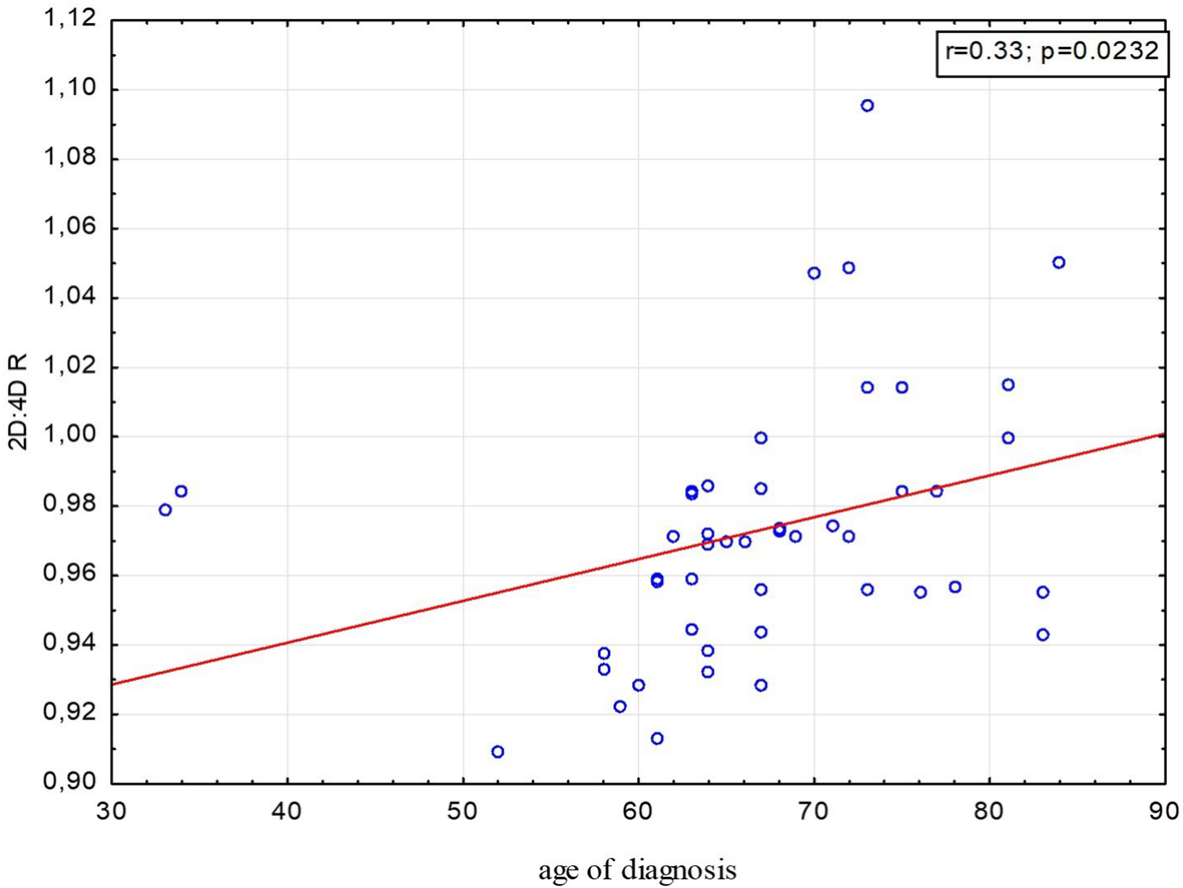
Positive correlation between age of lung cancer diagnosis and right 2D:4D in women.

Table 1 shows mean values of right and left 2D:4D for patients with lung cancer and control women and men. Logistic regression indicated that right 2D: 4D in the female group correlates with the risk of lung cancer. This correlation has a negative direction, i.e. higher values of right 2D:4D reduces, while lower right 2D:4D increases the likelihood of the disease in the female group (Table 2, Fig. 1). The model which included both variables: smoking and right 2D:4D gave the best classification accuracy: 83.3% for female patients with lung cancer and 82.4% for control women. Moreover, we found a correlation between right 2D:4D and the age of lung cancer diagnosis in women. In those with low right 2D:4D the onset of the disease was earlier than in women with higher 2D:4D (Table 3, Fig. 2). In men, 2D:4D was not associated with the risk of lung cancer (Table 4).

**Table 1:** Patients’ and controls’ right and left 2D:4D characteristics.

Group	n	2D:4D R		2D:4D L		Δ2D:4D	
		${\overline{\text{x}}}$	SD	${\overline{\text{x}}}$	SD	${\overline{\text{x}}}$	SD
Female patients (lung ca)	48	0.973	0.037	0.976	0.045	− 0.004	0.032
Control women	119	0.997	0.036	0.999	0.033	− 0.002	0.032
Male patients (lung ca)	61	0.97	0.04	0.977	0.042	− 0.006	0.034
Control men	78	0.978	0.037	0.979	0.031	− 0.001	0.022

${\overline{\text{x}}}$ mean, *SD* standard deviation.

**Table 2:** Assessment of the probability of lung cancer on the basis of the 2D:4D ratio in a group of women.

Predictor	Rating	Standard error	95% CI for rating		OR	95% CI for OR		p	R^2^ Nagelkerke
2D:4D R	− 15.79	7.48	− 30.45	− 1.13	1.38E−7	5.94E−14	3.21E−1	**0.035**	0.545
2D:4D L	− 4.48	6.95	− 18.09	9.14	0.01	1.39E−8	9.36E+3	0.52	0.521
Δ2D:4D	− 10.19	7.04	− 23.99	3.62	3.8E−5	3.8E−11	3.73E+1	0.148	0.528

Correlations of 2D:4D with the probability of lung ca were tested in separate models adjusted for smoking.

*OR* odds ratio, *p* probability for Wald test checking regression parameters’ relevance, *R*^*2*^* Nagelkerke* a measure of model fit to the data. Bold font indicates statistical significance.

**Table 3:** Correlation between 2D:4D and the age of lung cancer diagnosis.

Correlations	Women		Men	
	r	p	r	p
2D:4D R × age of diagnosis	**0.33**	**0.023**	− 0.01	0.966
2D:4D L × age of diagnosis	0.21	0.155	0.06	0.634
Δ2D:4D × age of diagnosis	0.09	0.558	− 0.08	0.523

Bold font indicates statistical significance.

**Table 4:** Assessment of the probability of lung cancer on the basis of the 2D:4D ratio in a group of men.

Predictor	Rating	Standard error	95% CI for rating		OR	95% CI for OR		p	R^2^ Nagelkerke
2D:4D R	− 2.30	6.49	− 15.0	10.43	1.00E 1	2.98E−7	3.38E+4	0.723	0.509
2D:4D L	4.54	7.350	− 9.86	18.95	93.97	5.21E−5	1.70E+8	0.536	0.504
Δ2D:4D	− 7.65	7.82	− 22.98	7.69	4.8E−4	1.1E−10	2.18E+3	0.328	0.51

Correlations of 2D:4D with the probability of lung ca were tested in separate models adjusted for smoking.

*OR* odds ratio, *p* probability for Wald test checking regression parameters’ relevance, *R*^*2*^* Nagelkerke* a measure of model fit to the data.

We also considered whether 2D:4D differs among women and men with different histological types of lung cancer (small-cell lc vs. non-small-cell lc). Only Δ2D:4D in men was lower in the case of small-cell lung cancer in comparison to non-small-cell lung cancer (Table 5).

**Table 5:** Histological type of lung cancer and digit ratios in women and men.

Ratio	Small-cell lc n = 11		Non-small-cell lc n = 37		df	*t* test		Effect size
	${\overline{\text{x}}}$	SD	${\overline{\text{x}}}$	SD		t	p	Hedge’s g
Women								
2D:4D R	0.98	0.032	0.971	0.039	46	0.76	0.451	0.26
2D:4D L	0.984	0.041	0.974	0.046		0.64	0.527	0.22
Δ2D:4D	− 0.004	0.032	− 0.004	0.033		− 0.01	0.992	< 0.01

Ratio	Small-cell lc n = 14		Non-small-cell lc n = 47		df	*t* test		Effect size
						t	p	Hedge’s g
Men								
2D:4D R	0.97	0.064	0.970	0.032	59	− 0.01	0.989	< 0.01
2D:4D L	0.996	0.056	0.971	0.036		1.54	0.142	0.59
Δ2D:4D	− 0.026	0.043	− 0.001	0.029		− **2.47**	**0.016**	**0.74**

*Hedge’s g* a corrected effect size measure. Bold font indicates statistical significance.

### Smoking and 2D:4D

Among female and male patients with lung cancer most were smokers: 91.7% and 91.8% respectively. Due to the small number of non-smokers in the patients’ group no further analysis was done. In controls 21.8% women and 26.7% men were smokers. We did not find a correlation between smoking and right 2D:4D in control women (*F* = 2.23; df = 1; *p* = 0.138) and men (*F* = 0.36; df = 1; *p* = 0.55). However, left 2D:4D in women smokers (*F* = 13.91; df = 1; *p* < 0.05) but not in men, (*F* = 0.31; df = 1; *p* = 0.577) was lower than in female non-smokers.

## Discussion

2D:4D may be useful in understanding the aetiology of sex steroid-dependent cancers. However, there is also the possibility that 2D:4D may aid in the prediction of susceptibility and progression of the disease, particularly where the inhibition of sex steroids may be part of the treatment ^28^. Our results showed that women with lung cancer have lower 2D:4D compared to controls and that among these women, age at diagnosis is positively related to 2D:4D. In men with lung cancer we found fewer significant effects but it is of interest that those with small-cell lung cancer have low Δ2D:4D compared to those with non-small cell lung cancer.

A recent meta-analysis by Bunevicius found evidence that that the prenatal sex hormone environment may influence susceptibility to develop some cancers later in life. Low 2D:4D may be associated with prostate cancer and brain tumours and high 2D:4D with risk of breast cancer, younger age of presentation of breast cancer, cervical dysplasia and brain tumours. There was no evidence that testicular cancer, gastric cancer, and oral cancer were associated with 2D:4D^33,40^.

In the study concerning 2D:4D association with gastric cancer in Chinese women the authors predicted that low 2D:4D would be associated with higher cancer risk. This prediction was based on the observation that oestrogen may inhibit the development of gastric cancer^41,42^. In our study we hypothesize that prenatal oestrogens may play a protective role against lung cancer in adult life. This may be due to maturation of lungs during foetal development which is stimulated by oestrogens and inhibited by testosterone^36,37^. In support of this, adult lung function has been reported to be positively related to 2D:4D. Park et al. (2014) found a positive relationship between right hand 2D:4D and pulmonary function test findings. In males, 2D:4D was positively related to forced vital capacity and forced expiratory volume in 1 s. In male smokers, lung functions were correlated with smoking exposure rather than 2D:4D. In female never-smokers, lung functions were positively correlated with 2D:4D. Park et al. (2014) concluded patients with lower 2D:4D tended to have decreased lung function^43^.

In contrast to our finding that lung cancer in women and small-cell lung cancer in men is associated with low 2D:4D or low Δ2D:4D, Manning and Fink have reported that high 2D:4D was linked to smoking^44^. Smoking is a major causative factor in lung cancer so the association of smoking choice with high 2D:4D seems in contradiction to our finding of a link between low 2D:4D and lung cancer. The explanation may lie in the effect of smoking on circulating testosterone levels. There is evidence that cigarette smoking leads to an acute increase in testosterone in men and women^45^. The effect may in part arise because cotinine, a tobacco metabolite, inhibits testosterone breakdown. However, other factors may apply here because a range of physiological and behavioural “challenges” may stimulate spikes in testosterone. The magnitude of such spikes has been linked to 2D:4D, such that low right 2D:4D and low Δ2D:4D individuals produce marked increases in testosterone^46^. Exogenous testosterone supplementation to increase strength and appearance has been reported to be common in men with high 2D:4D^47^. High 2D:4D, smoking and testosterone supplementation may be associated through a drive to increase circulating testosterone by men with high 2D:4D. Moreover, these effects may be moderated by lung efficiency such that high 2D:4D individuals may be able to tolerate the negative effects of smoking more readily than low 2D:4D individuals.

In our study we found that women with lung cancer had low right 2D:4D and also low right 2D:4D was associated with earlier onset of the disease. Such correlation was not present in male patients. Additionally, all women with squamous cell and small cell lung cancers were smokers. This may support an idea that women with low 2D:4D (lower prenatal oestrogens and/or lower sensitivity of oestrogen receptors) are more prone to cancer due to: decreased lung function, linked to developmental inhibition of the respiratory system, so more “vulnerable” lungs and/or less protective function of oestrogens on lung epithelium. As such our finding is consistent with that of Park et al.^43^ who reported that low 2D:4D was associated with compromised lung function. Such susceptibility combined with smoking may be an important risk factor of lung cancer. On the other hand, a possible protective role of circulating oestrogens in lung cancer risk is controversial and is still under discussion, so the hypothesis needs further verification. However, sex-dependence of lung cancer has already been raised by Manning who provided hypotheses for such correlations and possible pathophysiological explanations^48^.

There are some limitations to this study. The first is the size of the sample—although 61 male and 48 female patients seemed enough to create a classification model—there were not enough participants to externally validate the predictive value of the model on “new” cases. This means that it is difficult to evaluate the true predictive value of right 2D:4D in lung cancer in women. Additionally, focusing on specific lung cancer types inevitably limited the number of participants. Thus, our observation concerning masculinized Δ2D:4D among men with the most aggressive form of lung cancer (small-cell lung cancer) should be verified in further studies. This finding, although characterized with medium/strong effect size, needs to be confirmed in a larger study. We think an international collaborative study is desirable. This should include different ethnicities and frequencies of smoking. This would be helpful in verifying 2D:4D as a risk factor of lung cancer and early presentation of the disease. A further limitation may be related to the age of the controls, they were on average 12 years younger than patients with lung cancer. However, age differences did not affect anthropometric measurements (2D:4D is stable over lifetime and is not associated with adult sex hormones)^49–51^. Finally, due to the characteristics of our population we included only White participants so our conclusions may apply only to this group.

In conclusion, we have found that: (i) women with lung cancer have lower (more masculinized) 2D:4D compared to controls, (ii) among women with cancer, age at diagnosis is positively related to 2D:4D, i.e. women with masculinized 2D:4D present earlier with the cancer than women with feminized 2D:4D, (iii) among men with lung cancer, those with the most aggressive form (small-cell lung cancer) have masculinized Δ2D:4D (high prenatal testosterone and low prenatal oestrogen) compared to those with the less aggressive form (non-small cell lung cancer) (medium/strong effect size). In summary, our data suggests that masculinized right 2D:4D and Δ2D:4D are associated with a predisposition to lung cancer and/or the more aggressive forms of lung cancer in both women and men. These correlations cannot be explained by the relationship of 2D:4D with smoking.
